# Determinants of knowledge translation from health technology assessment to policy-making in China: From the perspective of researchers

**DOI:** 10.1371/journal.pone.0190732

**Published:** 2018-01-04

**Authors:** Wenbin Liu, Lizheng Shi, Raymond W. Pong, Hengjin Dong, Yiwei Mao, Meng Tang, Yingyao Chen

**Affiliations:** 1 Key Lab of Health Technology Assessment (Ministry of Health), Collaborative Innovation Center of Social Risks Governance in Health, School of Public Health, Fudan University, Shanghai, China; 2 School of Public Health and Tropical Medicine, Tulane University, New Orleans, Louisiana, United States of America; 3 Centre for Rural and Northern Health Research, Laurentian University, Sudbury, Ontario, Canada; 4 Centre for Health Policy Studies, School of Medicine, Zhejiang University, Hangzhou, Zhejiang, China; TNO, NETHERLANDS

## Abstract

**Background:**

For health technology assessment (HTA) to be more policy relevant and for health technology-related decision-making to be truly evidence-based, promoting knowledge translation (KT) is of vital importance. Although some research has focused on KT of HTA, there is a dearth of literature on KT determinants and the situation in developing countries and transitional societies remains largely unknown.

**Objective:**

To investigate the determinants of HTA KT from research to health policy-making from the perspective of researchers in China.

**Design:**

Cross-sectional study.

**Methods:**

A structured questionnaire which focused on KT was distributed to HTA researchers in China. KT activity levels in various fields of HTA research were compared, using one-way ANOVA. Principal component analysis was performed to provide a basis to combine similar variables. To investigate the determinants of KT level, multiple linear regression analysis was performed.

**Results:**

Based on a survey of 382 HTA researchers, it was found that HTA KT wasn’t widespread in China. Furthermore, results showed that no significant differences existed between the various HTA research fields. Factors, such as attitudes of researchers toward HTA and evidence utilization, academic ranks and linkages between researchers and policy-makers, had significant impact on HTA KT (p-values<0.05). Additionally, collaboration between HTA researchers and policy-makers, policy-relevance of HTA research, practicality of HTA outcomes and making HTA reports easier to understand also contributed to predicting KT level. However, academic nature of HTA research was negatively associated with KT level.

**Conclusion:**

KT from HTA to policy-making was influenced by many factors. Of particular importance were collaborations between researchers and policy-makers, ensuring policy relevance of HTA and making HTA evidence easier to understand by potential users.

## Introduction

Health technology assessment (HTA) has received much attention by clinicians, researchers and policy-makers in various health-care systems [1,2]. It has emerged as an important tool for evidence-based policy and clinical decision-making that comprehensively assesses safety, efficacy, cost-effectiveness and ethical concerns in utilizing health technology. In recent years, many HTA research units have been established around the world and a large number of HTA studies have been carried out.

However, conducting HTA studies is one thing, using HTA evidence to influence policy- or decision-making is another. For instance, in China, one of the largest developing countries, although technology assessment was a responsibility of the Department of Science and Education of the Ministry of Health (MoH) and many other administrative entities, HTA has little or no impact on most health technology-related decisions, though there is some evidence that HTA findings have influenced policy decision-making in such areas as gamma knife technology and prenatal diagnosis technology [3,4]. For HTA to be more policy relevant and for health technology-related decision-making to be truly evidence-based, promoting knowledge translation (KT), namely, the “exchange, synthesis and ethically-sound application of knowledge—within a complex system of interactions among researchers and users” [5], is a necessary first step [6].

Some studies have been conducted in other countries to measure KT and explore its determinants. For example, Peltz has categorized how scientific knowledge is used in public policy into three types, namely, symbolic utilization, conceptual utilization, and instrumental utilization. Symbolic utilization refers to justifying existing policies or positions of organizations (e.g., government). Conceptual utilization refers to changing one’s thinking but not necessarily one’s particular actions. Instrumental utilization refers to research findings being utilized as a basis for action, such as shaping policies and guiding implementation [7]. Going one step further, Estabrooks has developed measurement scales to separately measure each type of use [8].

Additionally, there are studies on the determinants of KT in general. Some studies have focused on researchers’ characteristics, such as their academic ranks fields of study and attitudes toward KT [9–14]. Others have focused on the research project itself and have explored the influence of timeliness, practicality and policy-relevance of research on KT [15–17]. It has also been suggested that efforts to make reports more readable and easier to understand would enhance adoption of research-based knowledge [18]. Finally, the importance of relationships between researchers and knowledge users, which include communication and collaboration during the research process, has also been examined in previous studies [19–21].

There are also studies more specific to HTA KT. For instance, studies have shown that providing timely information that is in line with decision-makers’ priorities increases research knowledge utilization [22,23]. Using less technical language tends to facilitate research uptake [24]. Building partnerships with user groups also plays a beneficial role. Previous studies have also highlighted the importance of leadership and creating cooperative relationships between researchers and knowledge users [25–27].

These studies are useful in understanding HTA KT and identifying its potential influencing factors. However, most of these studies are about KT in clinical practice, mainly focusing on the knowledge users, such as clinical decision-makers and so on. There is a dearth of literature on HTA KT, especially from the perspective of HTA researchers. Researchers and decision-makers often differ in terms of values, language patterns, and time perspective. Furthermore, production of HTA knowledge is the starting point of the KT process and researchers are the ones responsible for undertaking the investigation. Thus, it is necessary to understand the researchers’ perspectives, their attitudes toward research, and what they think about researchers’ social obligations. This does not diminish the importance of the perspectives of HTA knowledge users. But as noted earlier, much of existing HTA KT research focuses on policy- and decision-makers, and not enough is known about HTA researchers. Although we have published an earlier article exploring the differences in HTA KT between policy-makers and HTA researchers based on the same study, it mainly included factors at the individual level and the effect of the factors at the organizational level still remained unclear [28]. Thus, this study is an attempt to address this knowledge gap.

Additionally, much of the research on HTA utilization and its KT determinants has been conducted in developed nations but how HTA is utilized and how HTA KT is conducted in developing countries and transitional societies remain largely unknown. Owing to differences between developed and developing nations in socioeconomic development and health system management, the determinants of KT from HTA to health policy-making may be very different. To avoid the pitfall of wholesale transfer of inapplicable experiences from developed nations, it is necessary to carry out relevant research in developing countries such as China, one of the largest developing countries. Findings about KT determinants in China will probably be applicable to other developing countries.

Therefore, to fill the knowledge gap, we have selected China as a case study, with a view to investigating KT determinants both at the individual and organizational level from HTA research to health policy-making from the perspective of researchers.

## Material and methods

### Ethics statement

The research was approved by the research ethics board of the School of Public Health, Fudan University (IRB Approval Number: #2012-11-0382).

### Design

A cross-sectional survey design was used to obtain data for the analysis.

### Fields of health technology assessment (HTA) research

Since health technology is defined by the World Health Organization as the “application of organized knowledge and skills in the form of devices, medicines, vaccines, procedures and systems developed to solve a health problem and improve quality of lives”[29], this includes the pharmaceuticals, devices, procedures and organizational systems used in health care. Thus, this study divided the fields of health technology assessment (HTA) research as follows, drug, devices & equipment, procedure, organizational systems and the others.

### Questionnaire development

The items of the questionnaire were generated through a multi-step process: 1) review of related survey tools and relevant research papers; 2) interview with HTA researchers (pilot study); 3) examination of draft questionnaire by the research group; 4) revision and validation.

Firstly, a pool of 29 items, which consisted of candidate items that measured the level of HTA-related KT activities and their determinants, was generated, primarily through review of relevant measures and related research literature. Secondly, ten HTA researchers were recruited and asked to complete the draft questionnaire, and then interviewed about its comprehensiveness, relevance and clarity of expression. Thirdly, the research group sorted and qualitatively analyzed the interview data obtained to examine whether each item of the questionnaire reflected the construct concept. Lastly, according to the results of the pilot study, the questionnaire was revised to improve its comprehensiveness, relevance and clarity of expression. Some notes were also added in the questionnaire to ensure the respondents understood what they needed to do and how to do it.

The finalized questionnaire contains the following sections. 1) Communication (including 6 items, such as communication with policy-makers for setting HTA research priorities, determining research methods or conceptual framework, implementation of HTA research, data analysis, HTA report development and dissemination of HTA findings); 2) Organizational support mechanisms (including 4 items: guidance, training, staff with KT expertise and incentive mechanism for KT involvement); 3) Organizational linkages (including 4 items: cooperation with the other HTA units, cooperation with policy-making departments, cooperation with organizations utilizing health technologies, such as hospitals, and cooperation with health technology manufacturers); 4) Attitude toward KT (including 4 items: willingness to transfer HTA knowledge to policy-makers, perceived importance of HTA knowledge transfer, perceived importance of HTA evidence to health policy-making, perceived value of utilizing HTA evidence in policy-making); 5) KT level (including 6 items: academic translation, nominal translation, cognitive translation, reference translation, adoption translation and application translation). Academic translation means that research knowledge is published in academic journals. Nominal translation means that research knowledge is transmitted to policy-makers by submitting the research report, etc. Cognitive translation means that research reports are read and understood by policy-makers. Reference translation refers to research knowledge having been cited as a reference by policy-makers. Adoption translation refers to the adoption of research knowledge by policy-maker to underpin policies. Application translation means that research knowledge has resulted in policy application by policy-makers. Additionally, researchers’ assessments of their own HTA research, such as scientific rigor, timeliness, practicality, relevance to policy-making and user-friendliness of the language used, were included in the questionnaire.

In the survey, 5-point Likert scales were used. Possible responses were: 1 = strongly disagree, 2 = moderately disagree, 3 = neither agree nor disagree, 4 = moderately agree, 5 = strongly agree; or 1 = very bad, 2 = fairly bad, 3 = intermediate, 4 = good, 5 = excellent; or 1 = never, 2 = seldom, 3 = occasionally, 4 = often, 5 = always. Some demographic and related characteristics of HTA researchers, such as sex, education, organizational affiliation, academic rank and sources of research funding support, were also collected.

### Survey participants

HTA researchers in China working at HTA research units (including agencies/units with HTA as part of their titles, as well as others with any HTA research or related activities) and conducting HTA on clinical, social, economic, and ethical issues related to the use of health technology constituted the research population.

### Recruitment and consenting procedures

Since there was not a complete and reliable list of HTA researchers in China that could be used as a sampling frame, snowball sampling technique was used to recruit survey participants. To reduce possible selection bias, the snowball sampling process began with a list of initial participants that were as diverse as possible and were also known to us as HTA researchers. We then asked each of them to identify others who they thought should also be surveyed. In order to generate as comprehensive list of potential participants as possible, this process was repeated till no more new potential HTA researchers were identified.

The questionnaires and informed consent forms were sent to the survey participants via email. The ones who agreed to participate in the survey were asked to sign the informed consent form and filled out the questionnaire online.

### Scoring KT level

As mentioned above, KT level was measured by six items: academic translation, nominal translation, cognitive translation, reference translation, adoption translation and application translation. A score for each item was obtained from the participant’s responses in a range from 1 to 5.

Each item was presumed to be tiered and built on the previous one. In other words, the six items are cumulative in nature and each successive item needs to be weighed more heavily as one moves from the previous one to the next one (by 1 for item 1 (academic translation), by 2 for item 2 (nominal translation), and up to 6 for item 6 (application translation)) to produce a summary score for KT level with a range from 21 to 105 for each respondent [30].

### Statistical analysis

Descriptive statistics were used to describe the demographic and other characteristics of the HTA researchers, as well as the nature and extent of KT activities. To compare the various HTA research fields with respect to KT level, we used one-way ANOVA, more specifically, the Duncan’s multiple range test, which compares the means for groups in homogeneous subsets. Principal component analysis was performed to provide a basis for combining some of the original variables into a smaller number of factors, where appropriate. Multiple linear regression analyses were then performed to investigate the determinants of KT level. It was first estimated for all the research fields combined. Then it was estimated separately for each research field, because it was hypothesized that KT level in different fields of HTA research was not necessarily explained by the same variables.

Statistical analyses were performed using SPSS/version 13.0 statistical software. Two-sided P-value less than 0.05 was considered to be statistically significant. Listwise deletion was applied to deal with missing data.

## Results

### Characteristics of respondents

We distributed 561 questionnaires and received 382 completed questionnaires, achieving a response rate of 68.1%. The 382 HTA researchers ranged in age from 24 to 76, with the majority in the 30–49 age group. Slightly less than half of the researchers were male. Most researchers had a doctoral (51.8%) or a master’s (38.8%) degree. Nearly 80% worked at universities. About 30% and 35% of them were associate professors and full professors, respectively. Regarding primary research funding sources, 55% of the researchers had funding support from the government. With respect to the nature of their HTA research, more than half focused on health systems assessment and approximately 30% focused on drug assessment (Table 1).

**Table 1:** Demographic characteristics of the respondents.

	Frequency	Percentage (%)
**Gender**		
Male	179	46.9
Female	203	53.1
**Age**		
<30	24	6.3
30–39	151	39.5
40–49	116	30.4
50-	60	15.7
Missing	30	8.1
**Education**		
Bachelor’s	36	9.4
Master’s	148	38.8
Ph.D.	198	51.8
**Academic ranks**		
Professor	134	35.1
Associate Professor	108	28.3
Lecturer	112	29.3
Teaching Assistant	16	4.2
Others	12	3.1
**Affiliation**		
University	301	78.8
Not university	81	21.2
**Main funding source**		
Government	210	55.0
Not government	159	41.6
Missing	13	3.4
**Fields of HTA research**		
Drugs	106	27.8
Equipment	46	12.0
Procedure	61	16.0
Systems	213	55.8
Others	78	20.4
Missing	1	0.3

Regarding activity level by type of KT activity, our results indicated that 30%~40% of the HTA researchers usually or always published their research findings in academic journals or submitted HTA results to policy-makers. On the other hand, about 8.6% indicated that they never engaged in such knowledge dissemination activities. As one moves from academic translation to application translation, the proportion of respondents indicating that they never undertook that KT activity increased. Nonetheless, about 15% of the respondents reported that their HTA findings always or usually led to applications (Table 2).

**Table 2:** Activity level by type of KT activity.

	Never (%)	Rarely (%)	Sometimes (%)	Usually (%)	Always (%)	Average on 1–5 scales (S.D.)
Academic translation	8.6	13.4	38.2	30.6	9.2	3.18 (1.06)
Nominal translation	8.9	17.8	42.9	24.6	5.8	3.01 (1.01)
Cognitive translation	8.1	19.1	44.5	25.1	3.1	2.96 (0.95)
Reference translation	10.7	17.3	49.5	20.9	1.6	2.85 (0.92)
Adoption translation	15.7	26.2	42.9	14.4	0.8	2.58 (0.95)
Application translation	16.0	26.2	41.9	15.2	0.8	2.59 (0.96)

A KT summary score was calculated for each researcher and the results of the Duncan’s test were reported. The means of the KT summary scores ranged from 51.06 for “others” to 61.72 for “systems”. The Duncan’s test appears to show that there were two homogeneous subsets of HTA research field. There was no statistically significant difference between “others” and “drugs” with respect to levels of KT activity. Likewise, there were no statistically significant differences between “drugs”, “procedures”, “equipment” and “systems” (Table 3).

**Table 3:** Means of knowledge translation for different HTA research fields in homogeneous subsets (Duncan’s test).

HTA research fields	Subset for α = 0.05		
	Number of observations	1	2
Others	78	51.06	
Drugs	106	56.21	56.21
Procedure	61		57.08
Equipment	46		60.96
System	213		61.72
Significance ^a^		0.057	0.061

^a^ When the significance test is above the threshold α = 0.05, the null hypothesis cannot be rejected.

The principal component analysis resulted in four factors with items of high loadings (0.50 or over). An examination of the variables associated with these four factors suggested they represented linkage mechanisms, attitudes, organizational support, and organizational linkages of HTA research units, which were similar to how our original questions were grouped. The factors, reliabilities, means and standard deviations of these variables were listed in Table 4.

**Table 4:** Factor analysis of scale on HTA knowledge translation and its items (n = 382).

Name of scale Items	Factor loading	Cronbach’s alpha	Possible range	Min-Max	Mean	SD
		0.880 (Overall)				
**Linkage mechanism**		0.930				
Communication with policymakers for setting priority of HTA research topic	0.831	-	1–5	1–5	3.36	0.97
Communication with policymakers for determining research methods or conceptual framework	0.827	-	1–5	1–5	3.08	0.96
Communication with policymakers for implementation of HTA research	0.869	-	1–5	1–5	3.24	0.94
Communication with policymakers for survey data analysis	0.854	-	1–5	1–5	3.30	0.97
Communication with policymakers for HTA report development	0.863	-	1–5	1–5	3.17	0.98
Communication with policymakers for HTA evidence dissemination	0.776	-	1–5	1–5	2.79	0.97
**Attitude toward HTA and KT**		0.672				
Willingness to transfer the HTA knowledge to policymaking	0.647	-	1–5	1–5	4.24	0.67
Perceived importance of transferring HTA knowledge	0.830	-	1–5	1–5	4.47	0.69
Perceived importance of HTA utilization in health policymaking	0.853	-	1–5	1–5	4.69	0.60
Expect value of utilizing HTA findings in policymaking	0.534	-	1–5	1–5	4.05	0.89
**Organizational support**		0.851				
Guidance for KT	0.881	-	1–5	1–5	2.09	0.94
Train for KT	0.836	-	1–5	1–4	2.20	0.91
Special staff for KT	0.751	-	1–5	1–5	2.48	1.01
Incentive mechanism for successful KT	0.693	-	1–5	1–5	2.40	1.04
**Organizational linkage of HTA research unit**		0.799				
Cooperation with the other HTA research units	0.772	-	1–5	1–5	3.42	0.78
Cooperation with policymaking department	0.715	-	1–5	1–5	3.48	0.87
Cooperation with organizations utilizing health technologies (Hospitals and so on)	0.799	-	1–5	1–5	3.51	0.89
Cooperation with organizations manufacturing health technologies	0.684	-	1–5	1–5	2.93	0.94
**Developing the HTA report in easy-to-understand language**	Single item	-	1–5	1–5	3.23	0.97
**Scientific rigor of the HTA report**	Single item	-	1–5	1–5	3.69	0.61
**Submission of HTA reports to the policymakers at the right time**	Single item	-	1–5	1–5	3.68	0.63
**Practicality of the HTA evidence**	Single item	-	1–5	1–5	3.73	0.70
**Relevance of HTA research to policy-making**	Single item	-	1–5	1–5	3.60	0.79

### Multivariate linear regression analysis

The regression analysis results were summarized in Table 5. In the comprehensive model that included all fields of HTA research, academic ranks, government as the main source of funding support, relevance to policy-making, attitudes, “making HTA report easier to understand”, “individual linkage mechanism”, “organizational support” and “organizational linkage” were significantly and positively related to KT level. Scientific rigor was negatively associated with KT level (P < 0.05). Other variables, such as sex, education, and organizational affiliation, were found to be unrelated to KT level. The total amount of variance explained by this model, as shown by the adjusted R^2^, was 0.50.

**Table 5:** Multivariate linear regression models for predicting KT levels of HTA.

	All		Drug		Device & Equip		Procedure		Systems		Others	
	B	Beta	B	Beta	B	Beta	B	Beta	B	Beta	B	Beta
Constant	50.379		43.113		-49.608		60.735		45.376		72.828	
Sex	-2.033	-0.060	-2.590	-0.082	-3.220	-0.095	0.465	0.013	-2.734	-0.083	-1.994	-0.058
Educate	-0.314	-0.012	0.853	0.035	7.601	0.281	-2.125	-0.072	-0.780	-0.031	-2.757	-0.115
Academic rank	2.759*	0.137	0.743	0.040	1.178	0.061	6.250*	0.281	2.842*	0.146	5.650*	0.269
Affiliation	-1.023	-0.025	-3.409	-0.085	3.866	0.107	0.421	0.011	-0.737	-0.019	-5.522	-0.124
Funding source	3.610*	0.105	8.084*	0.252	-0.039	-0.001	-3.226	-0.091	2.769	0.080	0.833	0.025
Policy-relevance	2.189*	0.102	-0.508	-0.025	3.964	0.153	1.603	0.084	2.625,	0.120	3.264	0.158
Scientific rigor	-2.943*	-0.106	2.572	0.091	2.884	0.086	0.985	0.034	-2.873,	-0.100	-2.187	-0.091
Timeliness	1.830	0.069	1.771	0.064	1.555	0.052	-2.248	-0.083	1.457	0.055	1.201	0.053
Practicality	1.891	0.078	-3.076	-0.124	3.674	0.122	4.855	0.223	4.259*	0.170	2.091	0.096
Easy to understand	2.096*	0.119	3.158*	0.200	3.593	0.224	0.496	0.027	2.248*	0.125	2.095	0.127
Individual linkage mechanism	8.379*	0.485	7.922*	0.484	6.801*	0.346	5.905*	0.345	7.852*	0.453	9.036*	0.502
Organizational support	2.592*	0.152	2.087	0.130	0.308	0.018	2.817	0.158	2.517*	0.154	2.259	0.130
Organizational linkage	2.242*	0.126	0.654	0.040	5.723,	0.255	6.903*	0.345	2.433*	0.122	3.442*	0.236
Attitude	1.741*	0.102	2.204	0.112	2.101	0.120	2.109	0.143	2.117*	0.122	0.541	0.035
N	369		104		46		59		213		73	
Adj R^2^	0.504		0.428		0.375		0.479		0.532		0.489	
F	27.714		6.494		2.926		4.809		18.202		5.919	
VIF(max)	2.149		1.762		3.297		5.181		1.949		3.885	

*Indicate that variable is significant at 5% level.

The next ten columns in Table 5 reported the regression results of the different fields of HTA research. The factor of individual linkage mechanism explained KT level in all fields of HTA research. The factor of organizational linkage and academic rank were significantly and positively associated with KT level in “procedures”, “systems” and “other” research field, but not in “drugs” and “device and equipment”. “Making HTA reports easier to understand” explained KT level in the research fields of “drugs” and “systems”, but not in “procedures” and “device and equipment”. Attitude toward KT, practicality of HTA evidence and organizational support were significantly and positively related to KT level for HTA research field of “systems”, but not for the other fields. As for government as the main source of funding support, it was significantly and positively related to KT level in HTA research field of “drugs”, but not in the other fields. The other six variables, namely, sex, education, affiliation, scientific rigor, submitting HTA reports to policy-makers at the right time and HTA relevance to policy-making were not found to be associated with KT level in all fields of HTA research. The total amount of variance explained in the research fields of “drugs”, “device and equipment”, “procedures”, “systems” and “others”, as shown by the adjusted R^2^, was 0.43, 0.38, 0.48, 0.53 and 0.49, respectively.

For linear regression models mentioned above, variable inflation factors (VIFs) were also computed for the independent variables to detect multi-colinearity. The maximum VIF among the independent variables was 5.18 (much smaller than 10), which indicated that multi-colinearity was not a significant problem [31].

## Discussion

This paper derived its dependent and independent variables from prior research on KT. Based on the responses from 382 HTA researchers, the findings of this study showed that, on the whole, between 30% and 40% of the HTA researchers surveyed usually or always had their research findings published in academic journals or submitted to policy-makers; but between 20% and 30% of the researchers did not even do the more basic KT tasks. Additionally, between 40% and 50% of the HTA researchers said that they rarely or never had their research findings adopted by policy-makers. These findings suggest that the application of HTA findings to policy-making is not widespread in China at present. Furthermore, comparisons of the means of KT summary scores show that no significant differences exist between the various fields of HTA research, such as “drugs”, “procedures”, “device and equipment” and “systems”. These findings prompted us to further explore whether the influencing factors of KT level were the same in different fields of HTA research. These questions were examined by using regression models.

The importance of collaboration between HTA researchers and policy-makers [18–20] was also highlighted in this study. Frequent exchanges between researchers and policy-makers during the processes of topic selection, research implementation, and result dissemination will enhance mutual understanding and trust. They may result in more targeted research questions and more useful research results. This suggests that more attention be paid to linkages between HTA researchers and policy-makers. However, such linkages often require time and other resources. Thus, it may be advisable to devise some incentive schemes to compensate HTA organizations and researchers for investing resources in establishing and maintaining linkage mechanisms [30].

Researchers’ characteristics, such as attitudes, academic ranks and research funding sources, have been shown to influence knowledge utilization in previous studies [8–13]. Similarly, in this study, attitudes and academic rank were shown to be good predictors in the comprehensive model and the model for the research field of healthcare systems. As the producers of HTA knowledge, HTA researchers’ attitudes, such as willingness to transfer their knowledge to users, their expectations of the use of HTA evidence in practice, may facilitate or hinder KT. Likewise, HTA researchers with higher academic ranks are likely to be more productive in research and publication, which may lead to greater utilization [8,9]. Additionally, funding support mainly from government is a good predictor of KT. Greater policy-relevance of studies funded by government may be a plausible explanation.

In line with previous studies conducted in other countries [14–16,21,22], we have found that policy-relevance of HTA research, practicality of HTA evidence and making research outcomes easier to understand influence KT level. Therefore, to enhance the impact of HTA, it is important for researchers to take policy-makers’ needs into consideration and make recommendations more “actionable”. Especially for those studies focusing on policy-making, there is a requirement to directly engage with policy-makers throughout the entire research process, tailor research programs and outputs to policy-makers’ needs, and adopt an appropriate communication format to make it easier for non-researchers to understand. These efforts may entail some costs, which may not usually be covered by research funds. Therefore, research funders or research users may consider compensating or even rewarding researchers who engage in appropriate KT activities.

An interesting result is that scientific rigor of HTA research was negatively related to KT. This seemingly counter-intuitive finding is particularly worth-noting because scientific rigor is always considered a priority in academia. It is possible that policy-makers are not so much opposed to scientific rigor in research as “alienated” by impenetrable technicalities and academic jargons in some HTA reports and most academic publications. While it is necessary to maintain scientific rigor in research, it is equally important to ensure that research dissemination be done in a user-friendly manner. Translating research outcomes into policy-making is a difficult balancing act between ensuring research relevance and preserving scholarly integrity. This point is also supported by previous studies [17,23,32].

The importance of leadership and creating an environment favorable to KT has been noted in previous studies [24–26]. Our findings show that support from research organizations, such as providing guidance for HTA KT, having KT specialists on staff, establishing an incentive mechanism to reward KT activities and so forth will greatly facilitate HTA KT. This is particularly important because the current academic incentive system in China (as well as in many other countries) tends to focus more on SCI paper publication than on encouraging cooperation between researchers and policy-makers in KT or policy uptake. Changing this academic incentive system and research culture will be a challenging undertaking.

The contribution of this study is two-fold. On the one hand, it contributes to the advancement of knowledge. Most empirical studies on HTA KT have focused on developed countries and on clinical practice. Our study has attempted to better understand the factors at both the individual level and the organizational level (such as organizational support, organizational linkage of HTA) that influence the process from HTA research to health policy-making in China. On the other hand, by exploring the determinants of HTA KT activities, this study has suggested ways for both researchers and policy-makers in China, and possibly in other developing countries, to make the best use of HTA with a view to promoting evidence-based policy- and decision-making.

Some limitations of this study should be noted. Firstly, social desirability biases cannot be ruled out in this self-report study. Secondly, owing to the absence of a complete listing of HTA researchers in China, we resorted to a snowball sampling technique, which is commonly considered to be a non-probabilistic sampling method. We took several measures to control potential biases in the selection of the study participants, but since about 30% of the approached HTA researchers did not return their questionnaires, the existence of some biases cannot be ruled out. For those willing to participate in this study were more likely to be aware of the importance of KT and more likely to engage in KT activities, the results of KT level and other indicators reported in this study may be more positive than real situation Thirdly, this paper looks at HTA KT solely from the researchers’ perspective, but KT inevitably involves at least two sets of actors—researchers and users of research.

## Conclusion

HTA is expanding rapidly in China, which is a positive development. But for HTA to be as effective as it should be, researchers need to pay equal attention to the transmission of research findings to potential users and the translation of research into policies, decisions, clinical practice, etc. Without this, HTA is just an academic exercise that is of little value to health care and has little impact on society.

Our study has shown that while many HTA researchers in China were aware of the importance of using research to support policy- and decision-making, HTA KT activities were not widespread. Also, although some HTA researchers were involved in KT to a greater or lesser extent, more effort is needed and such effort needs to be more systematic and targeted. On the other hand, successful KT relies not just on the endeavours of researchers, but also on the support of HTA organizations and research commissioning or funding agencies. Such support may take the forms of incentive mechanisms to encourage KT involvement, providing appropriate KT training to researchers, hiring KT specialists to work with researchers, including funding for KT activities in research grants, etc. As well, decision- and policy-makers need to reach out to researchers and be receptive to research evidence. This last aspect is not well understood in China at this time and more research is needed.

Our study is about the HTA KT situation in China, but our findings could have wider implications. Other developing nations, particularly those at developmental levels similar to that of China, could learn from China’s experience and design strategies that suit their own unique circumstances or meet their special needs.

## Supporting information

S1 File “S1_data.xls”. This file showed all the data underlying the findings described in this manuscript.
(XLS)

S2 File “S2_Questionnaire HTA KT.doc”. This file included the questionnaire applied in this study for data collecting.
(DOC)
