# Supplementary material for: Determinants of knowledge translation from health technology assessment to policy-making in China: From the perspective of researchers
Source: PLoS One. 2018 Jan 4;13(1):e0190732. doi: 10.1371/journal.pone.0190732 (PMC5754132; doi:10.1371/journal.pone.0190732)
Supplement: S2 File — This file included the questionnaire applied in this study for data collecting. (DOC) [file pone.0190732.s002.doc]

**Questionnaire for HTA researchers on knowledge translation from HTA research to policy-making**

To understand how HTA research impacts on health policy-making, we are conducting a survey using a questionnaire to understand your opinions on the roles knowledge translation plays in the relationship between HTA research and policy-making. This study has been approved by the ethical review committee of School of Public Health, Fudan University. It will take you a few minutes to complete this questionnaire. Taking part in this study is completely voluntary. The information you provide will only be used for scientific research, and we promise that your privacy will be strictly protected. You do not have to participate if you don’t want to. You may also leave the study at any time. If you leave the study before it is finished, there will be no adverse consequence and you will not lose any benefits to which you are otherwise entitled. To protect privacy, you will fill out the questionnaire anonymously. But we assume that if you fill out the questionnaire, you agree to participate in the survey. Thank you very much!

**1 Socio-demographic Characteristics**

1.1 Gender: _________ A. Male B. Female

1.2 Age: _________

1.3 Educational level _________

A. Primary school B. Junior high school

C. Senior high school or secondary school D. Associate degree

E. Bachelor’s degree F. Master’s degree

G. Ph.D. degree

1.4 Professional title: _________

A. Full professor or equivalent

B. Associate professor or equivalent

C. Lecturer or equivalent

D. Teaching assistant or equivalent

E. Others

1.5 Organizationl affiliation: _________

A. University B. Other affiliations

1.6 Main source of research funding support: _________

A. Government B. Other sources

1.7 Main HTA research field: _________

A. Drugs B. Equipment C. Procedures

D. Systems E. Others

**2 Linkage mechanism**

2.1 How often did you communicate with policymakers for setting priority of HTA research topic ? _________

A. Never B. Seldom C. Occasionally D. Often E. Always

2.2 How often did you communicate with policymakers for determining research methods or conceptual framework？ _________

A. Never B. Seldom C. Occasionally D. Often E. Always

2.3 How often did you communicate with policymakers for implementation of HTA research？ _________

A. Never B. Seldom C. Occasionally D. Often E. Always

2.4 How often did you communicate with policymakers for survey data analysis？ _________

A. Never B. Seldom C. Occasionally D. Often E. Always

2.5 How often did you communicate with policymakers for HTA report development？ _________

A. Never B. Seldom C. Occasionally D. Often E. Always

2.6 How often did you communicate with policymakers for HTA evidence dissemination？ _________

A. Never B. Seldom C. Occasionally D. Often E. Always

**3 Attitudes toward HTA and KT**

3.1 You are willing to transfer the HTA knowledge to policymaking. _________

A. Strongly disagree B. Moderately disagree C. Neither agree nor disagree

D. Moderately agree E. Strongly agree

3.2 It is important to transfer HTA knowledge. _________

A. Strongly disagree B. Moderately disagree C. Neither agree nor disagree

D. Moderately agree E. Strongly agree

3.3 It is important to utilize HTA evidence in health policymaking. _________

A. Strongly disagree B. Moderately disagree C. Neither agree nor disagree

D. Moderately agree E. Strongly agree

3.4 HTA findings has great value in policymaking. _________

A. Strongly disagree B. Moderately disagree C. Neither agree nor disagree

D. Moderately agree E. Strongly agree

**4 Organizational support**

4.1 How often did your organization provide guidance when you intend to transfer the HTA research findings to policymakers? _________

A. Never B. Seldom C. Occasionally D. Often E. Always

4.2 How often did your organization conduct professional training when you intend to transfer the HTA research findings to policymakers? _________

A. Never B. Seldom C. Occasionally D. Often E. Always

4.3 How often did your organization assign special staff to help you in knowledge translation from HTA research to policymakers? _________

A. Never B. Seldom C. Occasionally D. Often E. Always

4.4 How often did your organization reward you for successful knowledge translation from HTA research to policymaking? _________

A. Never B. Seldom C. Occasionally D. Often E. Always

**5 Organizational linkage of HTA research unit**

5.1 How was the cooperation between your HTA research unit and the other HTA research units? _________

A. Very bad B. Fairly bad C. Intermediate D. Good E. Excellent

5.2 How was the cooperation between your HTA research unit and policymaking departments? _________

A. Very bad B. Fairly bad C. Intermediate D. Good E. Excellent

5.3 How was the cooperation between your HTA research unit and organizations utilizing health technologies (hospitals and so on)? _________

A. Very bad B. Fairly bad C. Intermediate D. Good E. Excellent

5.4 How was the cooperation between your HTA research unit and health technology manufacturers? _________

A. Very bad B. Fairly bad C. Intermediate D. Good E. Excellent

**6 KT level**

6.1 How often did you publish your HTA research findings in academic journals? _________

A. Never B. Seldom C. Occasionally D. Often E. Always

6.2 How often did you submit your HTA research results to relevant policymakers? _________

A. Never B. Seldom C. Occasionally D. Often E. Always

6.3 How often do you think your HTA research reports is read and understood by the relevant policymakers? _________

A. Never B. Seldom C. Occasionally D. Often E. Always

6.4 How often were your HTA research findings cited as reference by relevant policymakers? _________

A. Never B. Seldom C. Occasionally D. Often E. Always

6.5 How often were your HTA research results adopted as evidence in decision-making by relevant policymakers? _________

A. Never B. Seldom C. Occasionally D. Often E. Always

6.6 How often did your HTA research findings lead to applications by relevant policymakers? _________

A. Never B. Seldom C. Occasionally D. Often E. Always

7. In general, how often do you prepare your HTA report to make sure it is easy to understand? _________

A. Never B. Seldom C. Occasionally D. Often E. Always

8. In general, how often do you submit your HTA report to policymakers expeditiously? _________

A. Never B. Seldom C. Occasionally D. Often E. Always

9. In general, how would you rate the scientific rigor of your HTA report ? _________

A. Very bad B. Fairly bad C. Intermediate D. Good E. Excellent

10. In general, how would you rate the practicality of the HTA evidence you submitted? _________

A. Very bad B. Fairly bad C. Intermediate D. Good E. Excellent

11. In general, how would you rate the relevance of your HTA research to policy-making ? _________

A. Very bad B. Fairly bad C. Intermediate D. Good E. Excellent

**That is all. Thank you for your participation!**
